# Relationship between White Matter Alterations and Pathophysiological Symptoms in Patients with Ultra-High Risk of Psychosis, First-Episode, and Chronic Schizophrenia

**DOI:** 10.3390/brainsci12030354

**Published:** 2022-03-07

**Authors:** Katarzyna Waszczuk, Ernest Tyburski, Katarzyna Rek-Owodziń, Piotr Plichta, Krzysztof Rudkowski, Piotr Podwalski, Maksymilian Bielecki, Monika Mak, Adrianna Bober, Błażej Misiak, Leszek Sagan, Anna Michalczyk, Jolanta Kucharska-Mazur, Jerzy Samochowiec

**Affiliations:** 1 Department of Psychiatry, Pomeranian Medical University in Szczecin, Broniewskiego 26 Street, 71-460 Szczecin, Poland; krudkowski@gmail.com (K.R.); piotr.podwalski@pum.edu.pl (P.P.); annakarolina6@wp.pl (A.M.); jola_kucharska@tlen.pl (J.K.-M.); samoj@pum.edu.pl (J.S.); 2 Department of Health Psychology, Pomeranian Medical University in Szczecin, Broniewskiego 26 Street, 71-460 Szczecin, Poland; ernest.tyburski@gmail.com (E.T.); katarzynarek90@gmail.com (K.R.-O.); piotrpp119@gmail.com (P.P.); maksbiel@gmail.com (M.B.); monika.mak@gmail.com (M.M.); 3 Institute of Psychology, University of Szczecin, Krakowska 69 Street, 71-017 Szczecin, Poland; adrianna.bober@usz.edu.pl; 4 Department of Psychiatry, Division of Consultation Psychiatry and Neuroscience, Wroclaw Medical University, 50-367 Wroclaw, Poland; mblazej@interia.eu; 5 Department of Neurosurgery, Pomeranian Medical University in Szczecin, Unii Lubelskiej 1 Street, 71-252 Szczecin, Poland; leszekm.sagan@gmail.com

**Keywords:** ultra-high risk, first episode psychosis, schizophrenia, diffusion tensor imaging (DTI), fractional anisotropy, white matter

## Abstract

Some symptoms of schizophrenia might be present before full-blown psychosis, so white matter changes must be studied both in individuals with emerging psychosis and chronic schizophrenia. A total of 86 patients—12 ultra-high risk of psychosis (UHR), 20 first episode psychosis (FEP), 54 chronic schizophrenia (CS), and 33 healthy controls (HC)—underwent psychiatric examination and diffusion tensor imaging (DTI) in a 3-Tesla MRI scanner. We assessed fractional anisotropy (FA) and mean diffusivity (MD) of the superior longitudinal fasciculus (SLF) and inferior longitudinal fasciculus (ILS). We found that CS patients had lower FA than FEP patients (*p* = 0.025) and HC (*p* = 0.088), and higher MD than HC (*p* = 0.037) in the right SLF. In the CS group, we found positive correlations of MD in both right ILF (rho = 0.39, *p* < 0.05) and SLF (rho = 0.43, *p* < 0.01) with disorganization symptoms, as well as negative correlation of FA in the right ILF with disorganization symptoms (rho = −0.43, *p* < 0.05). Among UHR individuals, we found significant negative correlations between MD in the left ILF and negative (*r* = −0.74, *p* < 0.05) and general symptoms (*r* = −0.77, *p* < 0.05). However promising, these findings should be treated as preliminary, and further research must verify whether they can be treated as potential biomarkers of psychosis.

## 1. Introduction

Schizophrenia is a debilitating disease with a poor prognosis. It affects many mental processes, resulting in a variety of disturbances in perception, thinking, cognitive functions, and multiple domains of functioning [1,2,3]. Most patients experience some level of reduced social motivation and difficulty with productivity or in performing daily living activities; this not only excludes them from the working community but also prevents them from making long-lasting social bonds and significantly decreases their quality of life [4]. It is also known to impose a great economic burden on society, mostly because of productivity losses associated with premature mortality [5]. Most common demonstrations of the disorder may be organized, after Timothy Crow, into two domains: (a) positive symptoms—additional or exaggerated mental processes, such as hallucinations or delusions; and (b) negative symptoms—meaning the absence of normal processes (i.e., apathy, poverty of speech, or flattened affect) [6]. Both dimensions can be measured and compared with different psychometric scales, such as, among others, the Positive and Negative Syndrome Scale (PANSS) [7], Scale for the Assessment of Positive Symptoms (SAPS) [8], Scale for the Assessment of Negative Symptoms (SANS) [9], Clinical Assessment Interview for Negative Symptoms (CAINS) [10], or Brief Negative Symptom Scale (BNSS) [11]. Given that schizophrenia affects multiple mental processes, other dimensions of symptoms can also be distinguished, as in a recent meta-analysis: positive symptoms (items: P1—Delusions, P3—Hallucinatory behavior, P5—Grandiosity, P6—Suspiciousness and persecution, G9—Unusual thought content), negative symptoms (items: N1—Blunted affect, N2—Emotional withdrawal, N3—Poor rapport, N4—Passive apathetic social withdrawal, N6—Lack of spontaneity, G7—Motor retardation, G16—Active social avoidance), disorganization (items: P2—Conceptual disorganization, N5—Difficulty in abstract thinking, N7—Stereotyped thinking, G5—Mannerisms/posturing, G10—Disorientation, G11—Poor attention, G13—Disturbance of volition, G15—Preoccupation), affect (items: G1—Somatic concern, G2—Anxiety, G3—Guilt feelings, G4—Tension, G6—Depression) and resistance (items: P4—Excitement, P7—Hostility, G8—Uncooperativeness, G14—Poor impulse control) [12].

First-episode psychosis (FEP) usually occurs in young adults and refers to people experiencing psychotic symptoms for the first time. Some symptoms, such as cognitive impairment, might be evident before the emerging psychosis and possibly even provide a prognosis of the course of the ultra-high risk (UHR) of psychosis state [13,14], but so far, the data are insufficient, and research on the UHR population is still ongoing. The UHR concept includes individuals experiencing subthreshold attenuated positive symptoms, transient symptoms of frank psychosis that do not meet the time criterion for schizophrenia, and family history of psychosis or schizotypal personality disorder accompanied by a significant decline in functioning. Retrospective studies show that even up to 73% of patients experienced some alarming symptoms a few years before their first admission to hospitals—usually depressive or negative symptoms [15]. After initial optimism about finally discovering the schizophrenia prodrome, the rates of transition to full-blown psychosis have been declining [16,17]. Still, because of the social impact of psychosis and many other comorbidities, from the increased risk of diabetes to greater risk of mortality [18], reducing the time from accurate diagnosis to adequate treatment seems crucial.

There are also structured questionnaires specifically aimed at this group of individuals that allow health professionals to assess and compare symptoms that may not yet be considered psychotic, but may somehow be prognostic of emerging mental health problems of different kinds, such as psychosis, affective disorders, or personality disorders [19]. The two most commonly used scales are the Structured Interview for Prodromal Symptoms (SIPS) [20] and the Comprehensive Assessment of At-Risk Mental States (CAARMS) [21], both divided into dimensions similar to PANSS: SIPS is divided into positive, negative, disorganized, and general/affective symptoms, while CAARMS is divided into positive symptoms, cognitive change (attention/concentration), emotional disturbance, negative symptoms, behavioral change, motor/physical changes, and general psychopathology. Many hypotheses have been proposed to explain the etiology of schizophrenia, among the most popular being the neurodevelopmental model proposed by Nancy Andreasen [22], which postulates the convergent influence of various factors (genes, viruses, nutrition, and psychological experiences) on brain development, leading to impairments in cognitive functioning and other symptoms. It has also been proposed that some symptoms might be due to disruptions of the white matter [23]. The most commonly mentioned areas are the prefrontal and temporal cortex, as well as their connections to other parts of the brain [24,25].

Constant improvement in neuroimaging techniques has given us some insight into white matter and might thus lead to a better understanding of the etiopathogenesis of schizophrenia spectrum disorders. One technique used to visualize the white matter architecture and integrity is diffusion tensor imaging (DTI). It is a non-invasive technique that enables us to map and graphically represent the plausible course of white matter bundles across the brain [26]. Based on the collected data, it is possible to make a 3D reconstruction of neural tracts known as a fiber tractography. In DTI-based tractography, a diffusion tensor is estimated for each voxel, and the main diffusion direction is computed as the major eigenvector of the tensor. Even though widely used, it is fundamentally limited in cases of complex fiber architecture (e.g., bending or crossing fibers) and might lead to errors in the attempted reconstruction of neural pathways or interpretation of DTI-derived metrics [27,28], which are often used to analyze the organization and integrity of white matter. These indices include, inter alia, fractional anisotropy (FA) and mean diffusivity (MD), with FA being the most commonly used. Although very sensitive to any changes in the diffusion in tissues, these metrics lack specificity, which makes analyzing them difficult [28]. To address these issues, the constrained spherical deconvolution (CSD) tracking algorithm can be used. It provides an estimation of a fiber orientation distribution (FOD), describing the number and orientation of fiber bundles passing through each voxel [29] and thus providing more accurate data.

Although data on parts of the brain affected in different stages of various mental disorders are still conflicting, meta-analyses and reviews of DTI studies on schizophrenia patients have shown significant reductions in FA in multiple brain areas, as presented in Table 1 [30,31,32], in the corpus callosum (CC), cingulum (CB), uncinate fasciculus (UF), left inferior longitudinal fasciculus (ILF), inferior fronto-occipital fasciculus (IFOF), superior longitudinal fasciculus (SLF), and others. Similar, but less robust, changes have been observed in FEP patients, especially among the CC, UF, ILF, SLF, IFOF, and among UHR individuals, in the SLF, ILF, IFOF, UF, CC, CB, among others, though single studies reported increased FA across a variety of areas [31,33].

Results about links between alterations among specific white matter bundles and the emergence of symptoms still seem inconsistent, but, as described above, they all refer to frontal and temporal lobes, which is consistent with structural changes and the dopaminergic dysregulation theory of schizophrenia and might explain at least some of its pathophysiology [34,35]. We therefore decided to pay closer attention to the SLF, which connects the frontal lobe with parietal lobes and the temporoparietal junction, and which plays a role in, inter alia, visuospatial functions, attention, motor control, working memory, and language-related processing and activities (understanding the content of heard or read text), as well as phonological processing [36]. There is existing evidence that disturbances in the SLF might be more common among patients prone to auditory hallucinations [37,38,39].

The ILF, on the other hand, connects the occipital lobe with temporal lobes and is involved in visual processes, such as object and face recognition, behavior related to vision, integrating visual and emotional processes, and visual hypoemotionality. It may help explain the pathophysiological basis for visual hallucinations [40,41], socio-emotional impairments [41], and delusions [42].

Surprisingly, there is still little research comparing white matter changes among patients from different points of the schizophrenia spectrum and analyzing the links between white matter integrity and various dimensions of psychopathology or severity of symptoms. Given the differences in methodology between studies, it is difficult to compile and evaluate the results; therefore, it is important to conduct research that includes all groups from this spectrum. Understanding the nature of emerging psychosis and its biological basis could lead to finding a biological marker of psychosis and to reducing the time of untreated psychosis—one of the most important prognostic factors. Faster diagnosis and treatment might significantly improve quality of life and reduce risks of stigmatization, isolation, and loss of employment. Considering the aforementioned limitations, we formulated the following objectives: the first aim of this study was to compare selected DTI indices (FA and MD) in the SLF and ILF in UHR individuals, patients with FEP, CS, and HC. The second aim was to assess whether there is any relationship between DTI indices and severity of symptoms. Based on the existing literature, we hypothesized that all groups would differ in terms of the level of white matter integrity, with the biggest alterations in the CS group. Our second hypothesis was that different dimensions of psychopathology would correlate with white matter disturbances in all groups and would vary with regard to severity of symptoms, allowing us to distinguish the groups along the schizophrenia spectrum.

## 2. Materials and Methods

### 2.1. Participants

A total of 86 unrelated patients—12 UHR individuals, 20 FEP patients, 54 chronic schizophrenia patients (CS), and 33 healthy participants (HC)—were recruited in the Clinic of Psychiatry of Pomeranian Medical University in Szczecin. The majority of patients were recruited during their hospitalization, and some of them through the inpatient clinic or day care unit. UHR diagnosis was based on SIPS: Attenuated Psychotic Symptoms (APS; 6 participants), Brief Limited Intermittent Psychotic Symptoms (BLIPS; 0 participants), Genetic Risk and Deterioration (GDR; 2 participants) individuals, as well as 4 who met the criteria for both APS and GDR individuals. FEP patients had clinical symptoms of psychosis with recent onset, no remission since the beginning of symptoms, and were diagnosed according to the International Statistical Classification of Diseases and Related Health Problems (ICD-10) [43] with schizophrenia F20 (16), acute and transient psychotic disorder F23 (3), or schizoaffective disorder F25 (1). The CS group included patients with a minimum of ten years illness duration and diagnosis confirmed with the use of Mini-International Neuropsychiatric Interview (MINI) [44].

The inclusion criteria were: being aged between 18–40 for UHR/FEP patients and 30–55 for CS patients; comprehension of the test procedure; ability to undergo a neuroimaging procedure; and a stable physical state. CS patients were considerably older, as we wanted to compare white matter integrity in later stages of the illness to assess the potentially neurodegenerative progression of the disease. Exclusion criteria included comorbid mental disorders, evident coincidence of symptoms with psychoactive substances, and severe somatic diseases or active inflammatory disorders. Because of the small sample size, we decided to include the results of one FEP patient aged 41.

All participants were examined by trained psychiatrists and underwent DTI and psychiatric assessment. In UHR/FEP patients, the complete examination was administered within four weeks of admission to the hospital or the outpatient clinic due to the frequency of lack of compliance in the acute phase of the disease.

The control group consisted of 33 healthy participants with no mental or neurological disorders, confirmed by both psychiatric evaluation and a structured self-report questionnaire, and was matched in terms of sex and years of education. The exclusion criteria were the same as for the patients group.

All participants gave written consent to participate in the study. The study protocol was approved by the local bioethics committee.

### 2.2. Clinical Assessments

UHR individuals were assessed by trained psychiatrists or psychologists with the use of SIPS—a questionnaire assessing positive, negative, disorganized, and general/affective symptoms. In SIPS, positive symptoms are rated with a score from 0 (absent) to 6 (severe and psychotic) on scales P1–P5 of the Scale of Psychosis-Risk Symptoms (SOPS). Ratings of 0–2 are in the normal range, 3–5 indicate a positive symptom that is considered in the risk syndrome range, and a score of 6 on one or more of P1–P5 scales indicates frankly psychotic symptom severity. Negative (N1–N6), disorganization (D1–D4), and general symptoms (G1–G4) are rated with a score from 0 (absent) to 6 (extreme). Each severity scale is followed by a space to provide a description of the symptom, including its beginning, duration, frequency, and impact on functioning. FEP and CS patients were examined by trained psychiatrists with PANSS. All groups were also administered the MINI questionnaire, to evaluate the diagnosis and exclude possible comorbidities, as well as the GAF [45], to assess the level of functioning. In order to compare symptom severity between SIPS and PANSS as closely as possible, we distinguished five dimensions on PANSS: positive symptoms, negative symptoms, disorganization, affect, and resistance, as suggested by Shafer and Dazzi [12]. All participants were treated according to their symptoms in accordance with good clinical practice and recommendations.

### 2.3. Image Acquisition

Participants underwent magnetic resonance imaging scanning using a 3-Tesla scanner (General Electric Signa HDxt, Milwaukee, WI, USA) at the Starmedica Imaging Centre in Szczecin. We acquired diffusion tensor images via single shot, echo planar imaging parameters as follows: repetition time (TR) = 11,675 s; echo time (TE) = 82.80 ms; numbers of excitation (NEX) = 2; matrix = 96 × 96; field of view = 240 mm × 240 mm; slice thickness = 3 mm; slice gap = 0.50; acquisition time = 10 min, 19 s, b value = 1000 s/mm^2^; diffusion gradient directions = 25.

### 2.4. Image Processing and Quality

The fiber tracking procedure, file conversion, and image preprocessing were performed with ExploreDTI software [46]. DICOM files were converted to the *.NIfTI format, in order to be compatible with the program. The converted images and the original data were checked for compatibility. Then, we corrected the data for signal drift, removed Gibbs Ring artifacts, and corrected artifacts due to motion and eddy current. The quality of the data was inspected visually. These data were used for obtaining whole brain tractography using the constrained spherical deconvolution tracking algorithm. Tracking parameters were set as follows: minimal FA for seed point selection (0.1)—0.2, maximal FA for seed point selection (0.1)—1, minimal FA to allow tracking (0.1)—0.2, maximal angle (0.90)—30, stem size (in mm)—1, minimal fiber length (in mm)—1, maximal fiber length (in mm)—500, seed point supersampling factor—(0 0 0), FA threshold—0.25 and the angular threshold—60 degrees. The ILF and SLF were visualized with an FA color map in the coronal plane. For visualization of the ILF, two regions of interest (ROIs) were used: ROI1 was in the temporal pole, and ROI2 was at the junction of the temporal and occipital lobes. For visualization of the SLF, ROI1 was at the pole of the frontal lobe just in front to the genu of the corpus callosum, and ROI2 was at the temporal stem. Then, parts of the tracts not anatomically involved in the ILF or SLF were excluded by creating regions of avoidance using the ROInot function. The FA and MD of the fiber tract were calculated automatically by the ExploreDTI descriptive statistics function.

**Figure 1:**
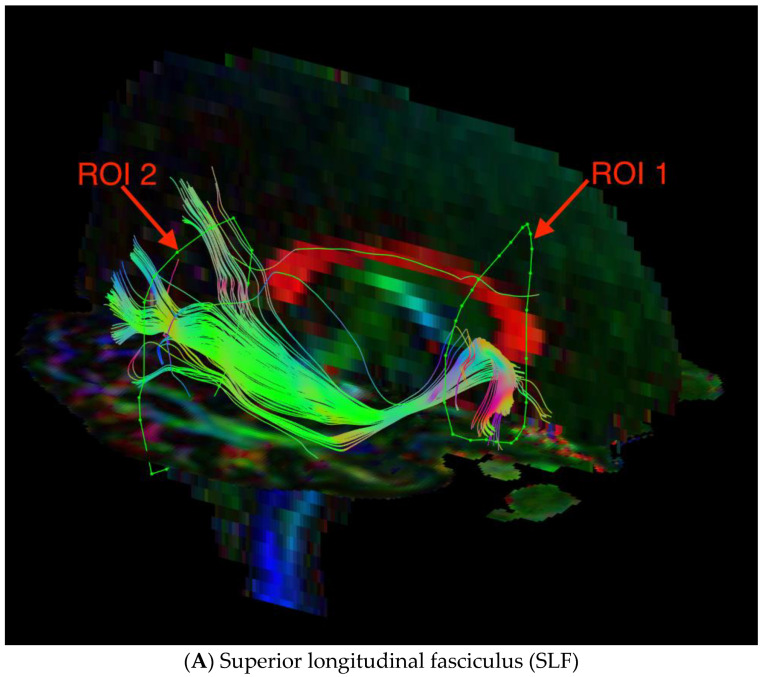

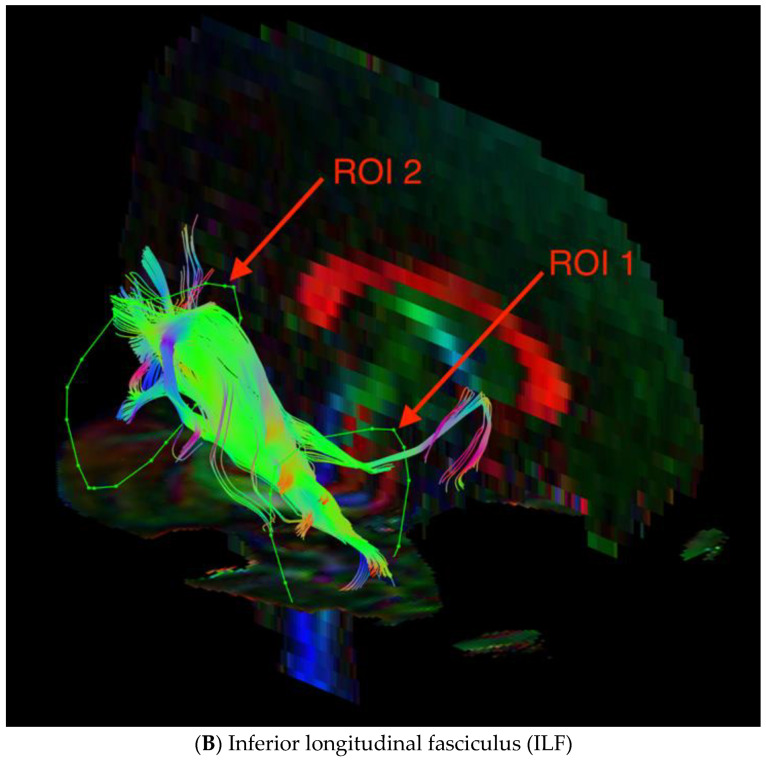
Diffusion tensor tractography of the (**A**) superior longitudinal fasciculus (SLF) and (**B**) inferior longitudinal fasciculus (ILF) with fractional anisotropy color maps (mid-sagittal plane). Green, red, and blue colors represent fibers running along the axis (anterior–posterior, left–right, and superior–inferior, respectively).

Images of the superior longitudinal fasciculus (SLF) and inferior longitudinal fasciculus (ILF) are presented below in Figure 1.

### 2.5. Statistical Analysis

We performed the statistical analysis with IBM SPSS 27 (IBM Corp., Armonk, NY, USA). Continuous variables are described in terms of means (*M*) and standard deviations (*SD*). We checked the normalities of the distributions using the Shapiro–Wilk test and skewness and kurtosis values. We assumed that skewness between −2 to +2 and kurtosis between −7 to +7 indicated normal distributions [47]. All groups had normal distributions of age, FA, and MD parameters for the ILF and for SLF. GAF global functioning was normally distributed for all three clinical groups, and psychopathological dimensions were normally distributed for UHR participants and FEP participants. Illness duration, chlorpromazine equivalent, and exacerbation were not normally distributed. When the relevant assumptions were met, differences between two groups were examined with Student’s *t* test, and when they were not, the Mann–Whitney *U* test was used. Differences between three groups were examined using one-way analysis of variance (ANOVA) when the relevant conditions were met, and the Kruskal–Wallis *H* test if not. The Games–Howell post hoc test (for parametric tests) and Dunn’s post hoc test (for non-parametric tests) were used for comparisons between groups. To determine the magnitudes of effect sizes for differences between groups, Cohen’s *d* or η^2^ (parametric tests) [48], and Wendt’s *r_U_* or and *E* (non-parametric tests) [49] were used. Subsequently, an analysis of covariance (ANCOVA) was performed to test for significant differences in FA and MD parameters. Age was used as a covariate since this variable might be related to DTI parameters. Lastly, in order to assess the relationship between measures of FA and MD and psychopathological symptoms in the three clinical groups, we estimated Pearson’s *r* or Spearman’s *rho* correlation coefficients. Holm–Bonferroni *p*-value correction was used for all statistical analyses (multiple comparisons and correlations). The alpha criterion level was 0.05, and there was a statistical power above 0.80 for all statistical analyses [48].

## 3. Results

### 3.1. Participant Characteristics

Demographic and clinical characteristics are presented in Table 2. There were no significant differences in sex; however, the groups differed significantly in age (*F*_(3, 115)_ = 23.54; *p* < 0.001; η^2^ = 0.38). Post hoc analyses showed that UHR and FEP groups were younger than patients with CS and HC (*p* < 0.001). There were significant differences between the three clinical groups in the value of chlorpromazine equivalent (*H*_(2, 8__4__)_ = 21.35; *p* < 0.001; *E* = 0.25), duration of illness (*H*_(2, 84)_ = 60.54; *p* < 0.001; *E* = 0.71), and exacerbation (*H*_(2, 84)_ = 43.04; *p* < 0.001; *E* = 0.51). Post hoc analysis showed that the UHR group had lower chlorpromazine equivalent than FEP patients (*p* = 0.019) and CS patients (*p* < 0.001). UHR individuals (*p* < 0.001 and *p* = 0.004) and FEP (*p* < 0.001 and *p* < 0.001) patients had shorter durations of illness and less exacerbations than CS patients, respectively. Moreover, there were group differences in type of medications: more CS patients had mixed (typical and atypical) medications and more UHR individuals had no treatment at all.

### 3.2. Differences in DTI Measures

**Figure 2:**
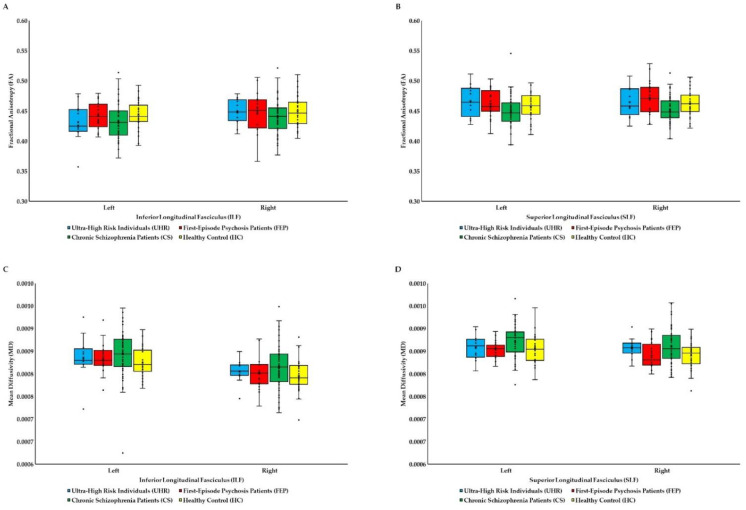
Fractional anisotropy (**A**,**B**) and mean diffusivity (**C**,**D**) of the inferior longitudinal fasciculus (ILF) and superior longitudinal fasciculus (SLF) for all groups. In all box plots, the bottom end of the box designates the first quartile, the line within the box indicates the median, and the top end of the box shows the third quartile. Whiskers indicate values 1.5 times the interquartile range below the first quartile and above the third quartile. Crosses represent average values. Circles designate individual observations.

As can be seen in Figure 2A,C, there were no significant differences between groups in FA and MD of the left and right ILF. However, there were significant differences between groups in the FA of the right SLF (*F*_(3, 11__5__)_ = 4.67; *p* = 0.004; η^2^ = 0.10) and the MD of the right SLF (*F*_(3, 115)_ = 3.26; *p* = 0.024; η^2^ = 0.07). Post hoc analysis showed that CS patients had lower FA than FEP patients (*p* = 0.025) and HC (*p* = 0.088; on trend toward statistical significance), and higher MD than HC (*p* = 0.037). Differences between all groups in the MD of the right SLF (*F*_(3, 114)_ = 3.69; *p* = 0.014; η^2^ = 0.09) did remain significant after co-varying for age but did not remain significant in the FA of the right SLF (*p* = 0.095). Post hoc analyses showed that CS patients had higher MD than HC (*p* = 0.040).

### 3.3. Differences in Psychopathological Dimensions

As can be seen in Table 3, FEP patients had greater severity of positive symptoms than CS patients (*Z* = −3.04; *p* = 0.01; *r_U_* = 0.45). After Holm–Bonferroni *p*-value correction (five corrections), there were no significant differences between these two clinical groups in the severity of the other four dimensions of psychopathological symptoms. Table 3 also shows the means of the severities of four psychopathological dimensions in UHR individuals.

### 3.4. Relationship between DTI Measures and Psychopathological Dimensions

**Figure 3:**
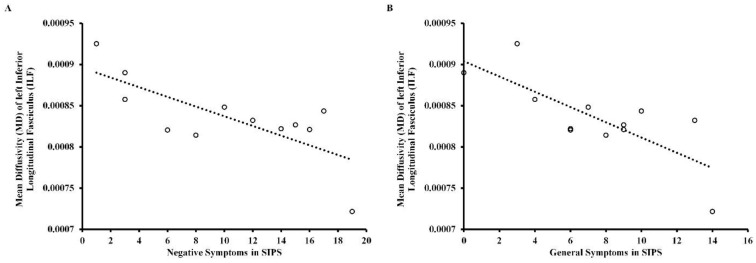
Scattergram for the relationship between: (**A**) mean diffusivity (MD) of the left inferior longitudinal fasciculus (ILF) and negative symptoms in SIPS and (**B**) mean diffusivity (MD) of the left inferior longitudinal fasciculus (ILF) and general symptoms in SIPS in ultra-high risk individuals (UHR). Circles designate individual observations.

**Figure 4:**
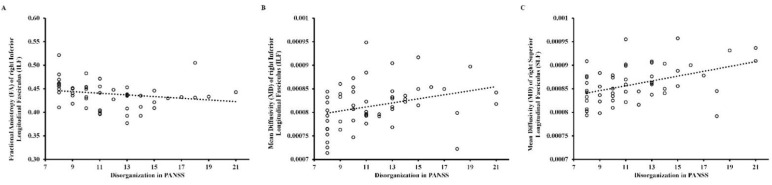
Scattergram for the relationship between: (**A**) fractional anisotropy (FA) of the right inferior longitudinal fasciculus (ILF) and disorganization in PANSS, (**B**) mean diffusivity (MD) of the right inferior longitudinal fasciculus (ILF) and disorganization in PANSS, and (**C**) mean diffusivity (MD) of the right superior longitudinal fasciculus (SLF) and disorganization in PANSS in chronic schizophrenia patients (CS). Circles designate individual observations.

Table S1 in the Supplementary Materials shows the correlations of FA and MD in the left and right SLF and ILF in the three clinical groups after Holm–Bonferroni *p*-value correction (four corrections for all DTI parameters). In UHR individuals, there were significant negative correlations between MD in left ILF and negative symptoms in SIPS (*r* = −0.74, *p* < 0.05) and general symptoms in SIPS (*r* = −0.77, *p* < 0.05), as depicted in Figure 3. In CS patients, there were significant positive correlations between MD in the right ILF (rho = 0.39, *p* < 0.05) and MD in the right SLF (rho = 0.43, *p* < 0.01) and disorganization symptoms, as well as a significant negative correlation between FA in right ILF (rho = −0.43, *p* < 0.01) and disorganization symptoms, as depicted in Figure 4. In FEP patients, there were no statistically significant correlations between DTI measures and psychopathological symptoms on PANSS.

## 4. Discussion

This study compared the two longest tracts of white matter—the superior longitudinal fasciculus (SLF) and inferior longitudinal fasciculus (ILF)—among ultra-high risk (UHR) individuals, patients with first episode psychosis (FEP), people with chronic schizophrenia (CS), and healthy controls (HC). We hypothesized that the groups would differ in terms of the most commonly analyzed DTI parameters—fractional anisotropy (FA) and mean diffusivity (MD). Our results revealed a reduction in FA in CS and FEP patients in the right SLF, which is in-line with previous research, though most of the studies showed either bilateral or left-sided reduction [50,51,52,53,54,55,56,57,58]. We also found higher MD in the right SLF among CS patients when compared with HC (statistical trend). In our research, we did not find any differences in the left SLF between the groups, which might be due to several reasons, such as small sample size, not enough participants with symptoms connected to this part of the tract (i.e., current auditory hallucinations), or the white matter developmental stage during the onset of symptoms [59]. No significant differences between the groups were found in either FA or MD in the ILF, which is in contrast with most studies [42,53,54,55,60,61] but might be due to the small sample size. Moreover, there are reports that young patients with schizophrenia do not differ in FA values and that the decline is only seen with increasing age [62] or that changes in FA in FEP were on a statistical trend-level only [63]. We did not find any significant anomalies in either FA or MD in any of the examined white matter tracts among the UHR group, which is not consistent with most studies but has been reported in some research [31]. These discrepancies may be partially explained by methodological issues, such as voxel-based vs tract-based DTI approaches or by the wide ranges of age and illness duration among patients. Some studies suggest that FA reduction might become more visible with increasing age, which could be caused by illness progression or pharmacological treatment [62,64]. Moreover, changes in DTI indices may potentially reflect different aberrations, such as axonal damage or quality of myelination [65]. There are consistent reports of reduced expression of genes (MAG, MAL, MBP, PLP, MOG, NRG1, and Olig2, among others) associated with oligodendrocytes and myelin, and therefore involved in neuronal development or signal transmission in schizophrenia [66,67]. These might lead to decreased intracortical connectivity and impaired information processing, resulting in some symptoms of schizophrenia—namely, cognitive decline, disorganized thinking, and impaired executive decision making [68].

The second aim was to assess whether there is any relationship between the DTI indices and severity of symptoms. Our research showed a significant negative correlation between MD in the left ILF and negative and general symptoms in SIPS, which, to our knowledge, is a novel finding—so far, only FA has been reported to correlate significantly with psychopathology, though the results were inconsistent both in terms of type of symptoms and specific white matter bundles [33]. It has been suggested that increased MD might be more sensitive to white matter damage and might appear before changes in FA, as suggested in some reports analyzing newly diagnosed patients with Parkinson’s disease (PD) [69]. A recent systematic review of PD patients showed a link between changes in MD in the ILF and some symptoms that are also observed in patients with schizophrenia spectrum disorders, such as cognitive decline, depression, and impaired facial emotion recognition [70], which underlines the need for further research on a larger group of patients.

We found no significant correlations between DTI measures and psychopathological symptoms in PANSS in the FEP group, which is in contrast with some previous reports that found a link between FA reduction in the left ILF among young patients with schizophrenia and worse overall symptom severity [53] or increased FA in the SLF and positive symptoms [71]. This may also be due to small sample size or the selection of the group—it has been suggested that some symptoms might be connected with specific white matter bundles, such as changes in the SLF with auditory hallucinations, so perhaps even though FEP patients showed more severe positive symptoms than the CS group in general, they did not meet the threshold for these particular symptoms to trigger the changes in the white matter [37,38,39].

In CS patients, there were three significant correlations: a positive relationship between (1) MD in the right ILF and (2) the right SLF and disorganization symptoms, and (3) a negative link between FA in the right ILF and disorganization symptoms. Changes in FA and MD in the ILF might, from the anatomical perspective, explain some of the disorganization symptoms, as the ILF is a part of the semantic ventral stream [41] and is partly responsible for lexical and semantic processes. Disruptions in the ILF have been reported in, inter alia, semantic dementia and primary progressive aphasia [41,72]. Some other disorganization symptoms, such as poor attention, memory, and motor control disturbances, might be caused by disruptions in the SLF, which represents the frontoparietal network (FPN) [36]. The FPN is crucial for activities of daily living, as it plays a role in tasks requiring executive control—executive dysfunctions are one of the key symptoms of schizophrenia [73]. Disrupted connectivity in the FPN in schizophrenia [74] has recently been described in a large meta-analysis, which seems to confirm the dysconnectivity hypothesis of the disease [23].

However interesting, our results should be treated with extreme caution for several reasons. The first reason being the small numbers of participants, especially in the FEP and UHR groups. Moreover, the sample sizes differed significantly, which might affect the robustness and statistical power of the results. We therefore used appropriate statistical tests (such as Kruskal–Wallis test, which does not require equal sample sizes) to minimize the effect of these differences. Additionally, we recruited only people treated in our facility, which means only individuals with high levels of personal distress. Notably, FEP patients had greater severity of positive symptoms than CS patients, which is probably due to the study design, as none of the CS patients were in the acute phase of the disease during the assessment. Unfortunately, the small size of the study groups prevented us from doing more complex analyses of possible confounding factors, such as duration of untreated psychosis, illness duration, or the effect of type and dosage of antipsychotic treatment. Therefore, more research is required.

Moreover, UHR individuals are a very heterogeneous group that, by definition, consists of persons with subthreshold attenuated positive symptoms, transient psychotic symptoms, genetic risk, and deterioration. These groups differ not only in terms of type and severity of symptoms, but also in transition risk [75], which might possibly alter the level of white matter disturbances [76]. Due to the scarcity of studies, especially those differentiating individuals who actually developed full-blown psychosis, it is impossible to draw any definite conclusions at this moment. During our research, only 2 UHR individuals transitioned into schizophrenia, which would not survive any statistical comparison. Moreover, taking into consideration the continuity and dynamics of brain development and different levels of white matter maturation between studied groups, a longitudinal approach seems necessary to fully illuminate DTI parameters in patients from across the schizophrenia spectrum and to verify the potential existence of a cause-and-effect relationship between white matter integrity and psychopathology or illness prognosis.

Another limitation of our study is that only the SLF and ILF were taken into consideration, so we cannot draw any conclusions about the global changes to fiber tracts. In addition, the limitations of diffusion magnetic resonance imaging should be mentioned. Due to the problem of the complex architecture of white matter and possible fiber crossing or bending, this method is biased towards Type II (false negative) errors, and some fibers might be excluded from the network. To address this issue, we applied the constrained spherical deconvolution (CSD) tracking algorithm, ensuring more accurate tract anatomy. On the other hand, CSD, as high angular resolution diffusion imaging, is more prone to Type I (false positive) errors with the inclusion of some aberrant fibers [77]. It also does not eliminate the partial volume effect (PVE)—namely, the possible contamination of the outer white matter voxel with its surrounding tissue (e.g., cerebrospinal fluid or gray matter) [27,78]. One way of dealing with PVE might be using the damped Richardson–Lucy algorithm that reduces spurious fiber orientations [79]. Moreover, to provide the most biologically plausible reconstructions of fiber tracts, anatomically constrained tractography (ACT), a method integrating anatomical knowledge with T1-weighted MRI to influence appropriate streamlines termination points, might be implemented [27]. Finally, low resolution of DTI images or artefacts may potentially lead to data misinterpretation. However preliminary, our results seem potentially useful, as they suggest directions for further research. Perhaps a wider approach and an observer-independent method such as tract-based spatial statistics could standardize the methodology, making it easier to compare results. In addition, other DTI measures, such as radial and axial diffusivity, should be analyzed. Additionally, taking into consideration the compartmentation of some tracts might be beneficial—in some studies the SLF has been divided into five subcomponents (SLF I–III, arcuate fasciculus, and temporoparietal SLF) [80], and there exist results linking FA reduction in the temporal component of the SLF to genetic predisposition to schizophrenia [81] or disturbances among the arcuate fasciculus to the severity of positive symptoms or specifically auditory hallucinations [82,83]. Focusing on the core symptoms of schizophrenia instead of groups of symptoms and paying more attention to cognitive deficits, which are widely described even before the onset of the disease [84], might also be beneficial in the future.

## 5. Conclusions

In conclusion, our study found decreased FA in the right SLF among CS and FEP patients and higher MD (statistical trend) in the right SLF in the CS group compared with HC, but no significant differences were found between any of the groups in either FA or MD in the ILF. As expected, in our study the most robust white matter disturbances were found in the CS group, less robust disturbances were found in the FEP group, while, surprisingly, no alterations were found in the UHR group. However, considering the aforementioned limitations, the results still support the concept of progressive changes among the schizophrenia spectrum disorders. Among the CS patients, correlations between MD in the right ILF and SLF and disorganization symptoms were found, as was a relationship between FA in the right ILF and disorganization symptoms. As disorganization has previously been linked to changes in neurocognition and poor functioning outcomes [85], those symptoms might be an important indicator of underlying white matter alterations and therefore considered as risk factors for a more severe course of illness. No significant correlations between DTI indices and psychopathological symptoms in PANSS were found in the FEP group, but our preliminary results suggest a possible link in the UHR group between MD in the left ILF and negative and general symptoms in SIPS. As suggested before, MD might be more sensitive to white matter disturbances than FA, requiring more attention. Further research on larger groups is necessary to verify these findings and assess whether they can be treated as a potential biomarker of psychosis. Comparing white matter alterations among the schizophrenia spectrum disorders will no doubt shed some light on the etiology of this debilitating disease. It is also recommended that the Shafer and Dazzi 5-factor PANSS scale be used in order to better capture symptom structure, as well as to improve compatibility between studies.

## Figures and Tables

**Table 1:** Comparison of FA between schizophrenia, first-episode psychosis, and UHR individuals [30,31,32].

	CS	FEP	UHR
↓FA	SLF, ILF, IFOF, CB, CC, UF, AF, IC, fornix, corona radiata, temporal lobe, occipital lobe, frontal lobe	SLF, ILF, IFOF, CC, UF, temporal lobe, parietal lobe, left frontal lobe	SLF, ILF, IFOF, CB, CC, UF, PTR, ATR, EC, IC, forceps minor, temporal lobe, frontal lobe
↑FA	Right frontal lobe, left occipital lobe, insula, IC, cerebellum, inter-hemispheric and cortico-cortical tracts		SLF, IFOF, UF, AF, ATR, forceps minor, frontal lobe, right fornix

Note: ↓—increased; ↑—decreased; FA = fractional anisotropy; CS = chronic schizophrenia patients; UHR = ultra-high risk for psychosis; SLF = superior longitudinal fasciculus; ILF = inferior longitudinal fasciculus; IFOF = inferior fronto-occipital fasciculus; CB = cingulum bundle; CC = corpus callosum; UF = uncinate fasciculus; AF = arcuate fasciculus; IC = internal capsule; EC = external capsule; ATR = anterior thalamic radiation; PTR = posterior thalamic radiation.

**Table 2:** Demographic and clinical characteristics of participants from four groups.

	Ultra-High Risk Individuals (UHR) (*n* = 12)	First-Episode Psychosis Patients (FEP) (*n* = 20)	Chronic Schizophrenia Patients (CS) (*n* = 54)	Healthy Control (HC) (*n* = 33)	*F*/*H*/*χ^2^*
Age: *M* (*SD*)	25.08 (4.81)	27.00 (5.43)	38.39 (6.64)	37.09 (8.08)	23.54 ^a,^***
Sex: female/male	6/6	13/7	23/31	20/13	4.27 ^c^
Antipsychotic medications:					
Atypical: *n* (%)	6 (50.00)	16 (80.00)	33 (61.12)	-	31.77 ^c,^***
Atypical and typical: *n* (%)	0 (0.00)	2 (10.00)	17 (31.48)	-	
Typical: *n* (%)	0 (0.00)	1 (5.00)	2 (3.70)	-	
No medications: *n* (%)	6 (50.00)	1 (5.00)	2 (3.70)	-	
Chlorpromazine equivalent (mg): *M* (*SD*)	131.58 (226.08)	504.90 (336.89)	632.09 (317.07)	-	21.35 ^b,^***
Duration of illness: *M* (*SD*)	1.07 (1.41)	0.39 (0.39)	15.18 (5.64)	-	60.54 ^b,^***
Exacerbation: *M* (*SD*)	4.08 (5.85)	1.10 (0.31)	6.37 (4.51)	-	43.04 ^b,^***
Global functioning in GAF: *M* (*SD*)	63.67 (14.20)	59.65 (17.25)	57.58 (15.24)	-	0.77 ^a^

GAF = Global Assessment of Functioning. ^a^ One-way analysis of variance *F* test. ^b^ Kruskal–Wallis *H* test. ^c^ Chi-squared test. *** *p* < 0.001.

**Table 3:** Psychopathological dimensions in participants from three clinical groups.

	Ultra-High Risk Individuals (UHR) (*n* = 12)	First-Episode Psychosis Patients (FEP) (*n* = 20)	Chronic Schizophrenia Patients (CS) (*n* = 54)	*Z*
Positive Symptoms in PANSS: *M* (*SD*)	-	11.40 (5.27)	7.59 (3.54)	−3.04 *
Negative Symptoms in PANSS: *M* (*SD*)	-	14.60 (5.40)	16.81 (6.57)	−1.33
Disorganization in PANSS: *M* (*SD*)	-	13.85 (4.57)	11.69 (3.47)	−1.89
Affect in PANSS: *M* (*SD*)	-	9.95 (3.89)	8.67 (3.39)	−1.51
Resistance in PANSS: *M* (*SD*)	-	5.85 (2.21)	4.61 (1.09)	−2.34
Positive Symptoms in SIPS: *M* (*SD*)	5.83 (3.90)	-	-	-
Negative Symptoms in SIPS: *M* (*SD*)	10.33 (6.09)	-	-	-
Disorganization in SIPS: *M* (*SD*)	3.83 (2.94)	-	-	-
General Symptoms in SIPS: *M* (*SD*)	7.42 (4.01)	-	-	-

PANSS = Positive and Negative Syndrome Scale. SIPS = Structured Interview for Psychosis-Risk Syndromes. * *p* < 0.05 (after Holm–Bonferroni *p*-value correction).

## Data Availability

Materials of the study reported here are available from the corresponding author on reasonable request.

## Supplementary Materials

The following supporting information can be downloaded at: https://www.mdpi.com/article/10.3390/brainsci12030354/s1, Table S1: Relationship between DTI measures in the Inferior Longitudinal Fasciculus (ILF) and Superior Longitudinal Fasciculus (SLF), and psychopathological symptoms in three clinical groups.
