# Supplementary material for: Relationship between White Matter Alterations and Pathophysiological Symptoms in Patients with Ultra-High Risk of Psychosis, First-Episode, and Chronic Schizophrenia
Source: Brain Sci. 2022 Mar 7;12(3):354. doi: 10.3390/brainsci12030354 (PMC8946295; doi:10.3390/brainsci12030354)
Supplement: Supplementary file 1 [file brainsci-12-00354-s001.zip › brainsci-1601258-supplementary.pdf]

Table S1. Relationship between DTI measures in the Inferior Longitudinal Fasciculus (ILF) and Superior Longitudinal Fasciculus (SLF), and psychopathological symptoms in three clinical groups.

	Inferior Longitudinal Fasciculus (ILF)				Superior Longitudinal Fasciculus (SLF)			
	Left		Right		Left		Right	
	FA	MD	FA	MD	FA	MD	FA	MD
Ultra-High Risk Individuals (UHR)								
	r	*r*	*r*	*r*	*r*	*r*	*r*	*r*
Positive Symptoms in SIPS	0.13	-0.14	0.15	-0.43	-0.16	-0.37	-0.03	-0.36
Negative Symptoms in SIPS	0.34	**-0.74 ***	0.61	-0.70	0.38	-0.61	0.43	-0.51
Disorganization in SIPS	0.20	-0.44	0.49	-0.39	0.03	-0.23	0.04	0.02
General Symptoms in SIPS	0.48	**-0.77 ***	0.53	-0.59	0.23	-0.55	0.23	-0.46
First-Episode Psychosis Patients (FEP)								
	r	*r*	*r*	*r*	*r*	*r*	*r*	*r*
Positive Symptoms in PANSS	0.22	0.08	0.36	0.00	0.15	0.17	0.21	0.00
Negative Symptoms in PANSS	0.06	-0.14	-0.18	-0.12	0.02	-0.02	-0.03	0.01
Disorganization in PANSS	0.05	0.31	0.15	0.20	-0.03	0.41	0.12	0.16
Affect in PANSS	0.56	-0.30	0.38	-0.34	0.52	-0.17	0.50	-0.27
Resistance in PANSS	-0.04	-0.21	0.15	-0.12	0.03	-0.18	0.06	-0.04
Chronic Schizophrenia Patients (CS)								
	rho	*rho*	*rho*	*rho*	*rho*	*rho*	*rho*	*rho*
Positive Symptoms in PANSS	0.02	0.06	-0.15	0.15	-0.15	0.19	-0.02	0.19
Negative Symptoms in PANSS	-0.10	0.06	-0.20	0.15	-0.09	0.08	0.03	0.10
Disorganization in PANSS	-0.27	0.32	**-0.43 ****	**0.39 ***	-0.17	0.22	-0.07	**0.43 ****
Affect in PANSS	-0.28	0.05	-0.36	0.12	-0.14	0.09	-0.28	0.14
Resistance in PANSS	-0.23	0.23	-0.20	0.22	-0.20	0.28	-0.28	0.47

PANSS = Positive and Negative Syndrome Scale. SIPS = Structured Interview for Psychosis-Risk Syndromes.
* $p < 0.05$. ** $p < 0.01$. (after Holm-Bonferroni *p*-value correction).
